# Mortality Among Patients With Invasive Group A Streptococcal Infections Caused by the M1_UK_ Lineage: A Retrospective Cohort Study in England and Wales

**DOI:** 10.1093/cid/ciaf492

**Published:** 2025-10-16

**Authors:** Ho Kwong Li, Nina Zhu, Olivia Waddell, Juliana Coelho, Roger Daniel, Rebecca L Guy, Theresa Lamagni, Shiranee Sriskandan

**Affiliations:** Centre for Bacterial Resistance Biology, Imperial College London, London, UK; Department of Infectious Disease, Imperial College London, London, UK; Department of Infectious Disease, Imperial College London, London, UK; Centre for Antimicrobial Optimisation, Imperial College London, London, UK; NIHR Health Protection Research Unit in Healthcare-Associated Infections and AMR, Imperial College London, London, UK; Department of Infectious Disease, Imperial College London, London, UK; NIHR Health Protection Research Unit in Healthcare-Associated Infections and AMR, Imperial College London, London, UK; Staphylococcus and Streptococcus Reference Section, AMRHAI, UK Health Security Agency, London, UK; Staphylococcus and Streptococcus Reference Section, AMRHAI, UK Health Security Agency, London, UK; Antimicrobial Resistance and Healthcare Associated Infections Division, UK Health Security Agency, London, UK; NIHR Health Protection Research Unit in Healthcare-Associated Infections and AMR, Imperial College London, London, UK; Antimicrobial Resistance and Healthcare Associated Infections Division, UK Health Security Agency, London, UK; Centre for Bacterial Resistance Biology, Imperial College London, London, UK; Department of Infectious Disease, Imperial College London, London, UK; NIHR Health Protection Research Unit in Healthcare-Associated Infections and AMR, Imperial College London, London, UK

**Keywords:** Streptococcus pyogenes, sepsis, mortality, lineage, M1_UK_

## Abstract

**Background:**

The M1_UK_ sublineage of *Streptococcus pyogenes* has driven recent post-pandemic surges in invasive group A streptococcal (iGAS) disease. We assessed case fatality rate (CFR) among patients with *emm1* iGAS in England and Wales, and then compared outcomes associated with M1_UK_ and ancestral *emm1* lineages.

**Methods:**

We linked *emm1* iGAS cases (December 2009–July 2022) with demographic and mortality records. Lineage was determined for isolates collected in 2010, 2013–2016, and 2020 via whole-genome sequencing or allele-specific PCR. Seven- and 30-day all-cause CFRs were estimated. Univariate and multivariate models assessed the association between lineage and risk of death.

**Results:**

Among 4952 *emm1* iGAS cases, lineage was assigned to 1356. The 30-day CFR was 24.4% for M1_UK_, 22.3% for M1_global_, 10.5% for M1_23SNP_, and 10.3% for M1_13SNP_. After adjustment for age and sex, lineage was not a significant predictor of 7- or 30-day mortality. Survival analysis showed rapid progression to death in both M1_UK_ and M1_global_ cases: 63.7% of deaths occurred within 1 day of diagnostic sampling. Among children under 15 years, 56.3% of fatal cases died before sampling, and 95.6% within 1 day of sampling.

**Conclusions:**

Mortality did not differ significantly between M1_UK_ and M1_global_ lineages, but more studies are required. Overall mortality from *emm1 S. pyogenes* remains strikingly high. The rapid time to death underscores the need for preventive measures and rapid diagnostic tools that act prior to culture-based confirmation, and highlights challenges for clinical trial design in iGAS.

## Teaser

*Emm*1 invasive Strep A incurs a high case fatality rate. In this national cohort, 24.4% of those with M1_UK_ iGAS died, though this was not different from ancestral *emm*1 lineages. Most deaths occurred within a day of samples being taken.

## Graphical Abstract

**Graphical Abstract:**
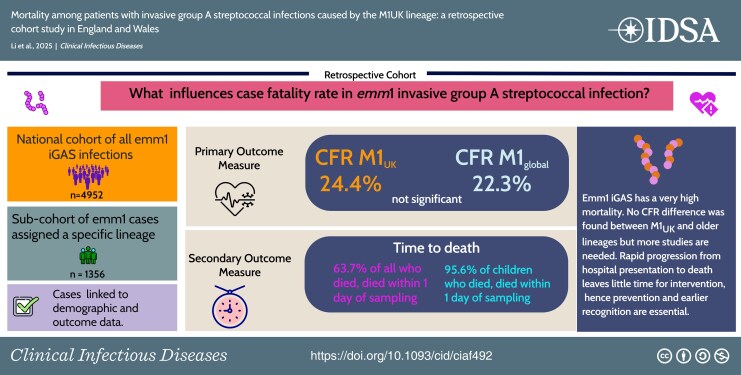
https://tidbitapp.io/institutional-portal/clinical-infectious-diseases/tidbits/mortality-among-patients-with-invasive-group-a-streptococcal-infections-caused-by-the-m1uk-lineage-a-retrospective-cohort-study-in-england-and-wales/update

Though rare, invasive group A Streptococcal (iGAS) infection can be associated with significant mortality and devastating sequelae for those affected. Manifestations include bacteremia, pneumonia, skin and soft tissue infection, including necrotizing fasciitis, puerperal sepsis, and streptococcal toxic shock [1, 2]. Since relaxation of non-pharmaceutical interventions (NPI) to combat the spread of severe acute respiratory syndrome coronavirus 2, upsurges in iGAS infections have been reported by national public health institutes and networks in Europe, North and South America, Australia, New Zealand, and Japan, some reporting high mortality [3–14]. Waning population immunity and consequent rebound upsurges of seasonal respiratory viruses were predicted following cessation of COVID-19-related NPI, although bacterial infections such as *Streptococcus pyogenes* were notably omitted [15]. Many, though not all, *S. pyogenes* upsurges have reported a predominance of an emergent lineage of genotype *emm*1, M1_UK_ [6–9, 14, 16, 17]. The M1_UK_  *S. pyogenes* lineage is distinguished from ancestral *emm*1 strains by 27 core SNPs and constitutive production of the erythrogenic streptococcal pyrogenic exotoxin A (SpeA) [18]. Emerging around 2008, M1_UK_ has almost replaced ancestral strains of *emm*1 in the United Kingdom representing 97% of *emm*1 [16], and currently represents a majority of *emm*1 isolates circulating in several European countries, Japan, Canada, New Zealand, and Australia [6–9, 11, 14, 16–21].

Mortality from iGAS is known to be influenced by individual host susceptibilities such as age and by causative strain genotype [2, 22]. *emm*1 and *emm*3 infections are acknowledged to be associated with increased risk of severe outcomes and mortality compared with other *emm* genotypes, although most large-scale population-based epidemiological studies were undertaken prior to iGAS being notifiable in England, and prior to the emergence of M1_UK_ [2, 22]. Invasiveness and virulence of *S. pyogenes* have been attributed in part to a propensity to undergo mutations in the two-component regulator covRS during the transition from non-invasive to invasive infection, resulting in de-repression of key virulence genes [23]. Although most experimental work relating to covRS has focused on *emm*1 [23], all *emm* genotypes are capable of similar mutations during clinical invasive infection [24]. Hence, it may be that other constitutive features unique to the modern clone of *emm*1, including M1 protein itself, the streptococcal inhibitor of complement (SIC), phage-encoded toxins and DNAses, and increased production of streptolysin O, are additionally important [25, 26].

Early reports of M1_UK_ association with increased iGAS frequency [18] and case severity [27], prompted us to review all deaths associated with *emm*1 iGAS, and to determine if the mortality associated with M1_UK_ iGAS differed from that associated with ancestral *emm*1 isolates. Ancestral isolates included the previously dominant “pandemic” M1_global_ strains, and 2 intermediate sublineages M1_13SNP_ and M1_23SNP_, which preceded the emergence of M1_UK_ [16]. We analyzed all *emm*1 iGAS cases in England and Wales that could be linked to outcome, and then analyzed a subset of *emm*1 iGAS cases where *emm*1 lineage had been assigned by previous genome sequencing or allele-specific PCR. To include sufficient iGAS cases associated with M1_global_ infection, we extended genotyping to include cases from 2010, while, to avoid any potential effect of population-level changes in anti-streptococcal immunity on case fatality, cases after 2020, including those during the upsurge (late 2022–2023), were not included in the lineage-based analyses.

## METHODS

### Demographic Data for Patients With *emm*1 iGAS 2009–2022

Demographic data (age, sex, ethnicity, and index of deprivation) and mortality of all patients who acquired invasive *emm*1 *S. pyogenes* infection between December 2009 and July 2022 in England and Wales were obtained by linking national bacteriology data held by United Kingdom Health Security Agency (UKHSA) to individual National Health Service (NHS) records. Sterile site *S. pyogenes* isolates are sent from all laboratories in England and Wales to the UKHSA *Staphylococcus* and *Streptococcus* Reference Section (SSRS); these isolates are characterized using *emm* gene sequencing. Patient records from Dec 2009-July 2022 with *emm*1 isolates were submitted to the NHS Demographic Batch Tracing Service (DBS) to identify date of death [28]. For unsuccessfully traced cases, a further assessment against the NHS Personal Demographics Service (PDS) was used to identify the date of death [29]. For mortality analysis, patients were considered lost to follow up if they could not be successfully traced to the NHS SPINE through either method. Demographic and ethnicity data were obtained through linkage to Hospital Episode Statistics (HES) using unique NHS number and date of birth [30]; the Index of Multiple Deprivation (IMD), a measure of relative socioeconomic deprivation for small areas in England, was derived from postcode. Ethnicity was allocated following the COVID-19 Health Inequalities Monitoring for England (CHIME) tool methodology [31]. UK Health Security Agency surveillance of infections for health protection purposes is approved under Regulation 3 of The Health Service (Control of Patient Information) Regulations 2020 and under Section 251 of the NHS Act 2006.

### Emm1 Lineage Assignment

Lineage assignment was undertaken for the *emm*1 iGAS isolates previously sequenced between 2013 and 2016 and for all iGAS *emm*1 from 2020 analyzed using allele-specific polymerase chain reaction (AS-PCR) [18, 32]. Lineage was assigned for all *emm*1 iGAS isolates from the year 2010 using AS-PCR [32] as part of this work.

### Statistical Analysis

We defined 30-day all-cause mortality to include a documented death date falling between 7 days before and 30 days after the specimen date (or 7 days before and 7 days after the specimen date for 7-day mortality) and calculated the case fatality rate accordingly, to accommodate the fact that some diagnostic samples were obtained postmortem. A linear trend analysis was performed with the sample year to detect whether there was a change in case fatality rate (CFR) over time.

Univariate logistic regression was performed to estimate odds ratio of 7-day and 30-day death for age, age group (under 15, 16 to 64, 65 to 84, 85 and above), sex (male, female), ethnicity (white, Asian, black, mixed, and other), and IMD quintile (1, most deprived, to 5, least deprived). Univariate analysis to determine the impact of lineage on predictors of 7-day and 30-day all-cause mortality was limited to those patients with *emm*1 iGAS for whom lineage was determined and where linkage to NHS data sources was successful. A multivariate model to analyze the impact of lineage was developed using variables identified as significant in the univariate analysis. All reported statistical tests were 2-sided, and *P*-values <.05 were considered statistically significant.

We performed survival analysis using specimen date as day 0, and a half-day interval to better differentiate those who died before a specimen was obtained (ie specimen obtained postmortem) from those who died on the same day as specimen date. As such, patients with a sample (that subsequently confirmed *S. pyogenes*) taken up to 7 days after death were assumed to have survived 0 days, while patients who died on the day that the sample was taken were assumed to have survived 0.5 days. Kaplan–Meier survival analysis was undertaken for the first 30 days after an *emm*1 isolate was identified, comparing age groups, and *emm*1 lineages [33] and compared using the log-rank test [34, 35]. As the study was limited by the national cohort size and existing genomic data, a sample size calculation was not undertaken.

## RESULTS

### Study Cohort, Isolates, and *emm*1 Lineages

From the whole study period December 2009–July 2022, 4952 of 5020 (98.6%) *S. pyogenes emm*1 iGAS isolates from England and Wales were successfully linked to patient ethnic group data in HES and survival/death information in DBS, after exclusion of 254 isolates that were not from England and Wales. (Supplementary Figure 1A).

For sub-analysis, 1445 *emm1* isolates were assigned a lineage; however, 67 of these were excluded as they were not from England and Wales. Of the 1378 invasive *emm*1 isolates assigned to a lineage, 98.4% (*n* = 1356) were linked to demographic and mortality data, accounting for 27.8% (1356/4952) of all *emm1* invasive isolates. These *emm*1 isolates comprised 39.1% M1_global_ strains (*n* = 530); 57.4% M1_UK_ strains (*n* = 778); 2.1% M1_13SNP_ strains (*n* = 29); and 1.4% M1_23SNP_ strains (*n* = 19). As expected, M1_UK_ strains became more frequent over time, accounting for 0.27% (1/375) eligible cases with lineage-assigned in 2010, and 90.1% in 2020 (201/223) (Supplementary Figure 1B). Three-quarters (203/268) of eligible *emm*1 isolates from 2020 were from the first three months of the year, prior to the main impact of the COVID-19 pandemic. (Supplementary Figure 1A).

### Case Fatality Rate

For the whole study period from December 2009 to July 2022, 1043 deaths were recorded within 30 days among the 4952 *emm*1-infected iGAS patients, yielding a 30-day CFR of 21.1% (95% CI: 19.9%, 22.2%). The 7-day CFR was 18.2% (95% CI: 17.1%, 19.3%) (Table 1). The 30-day CFR rose markedly with age (Supplementary Table 1). No significant change in the trend of CFR due to *emm*1 between 2009 and 2022 was observed (30-day CFR: *P* = .072, 7-day CFR: *P* = .140).

**Table 1.** Patient Characteristics, Univariate and Multivariate Analysis

Univariate		Number	Percentage (%)	Mortality, 30 d, odds ratio (95% CI)	*P-v*alue	Mortality, 7 d, odds ratio (95% CI)	*P-v*alue
Total	…	4952	…	…		…	…
Sex	Male	2608	52.67	Reference		Reference	…
	Female	2317	46.79	1.16 (1.01, 1.33)	.04	1.16 (1.01, 1.34)	.04
	Unknown	27	0.55	…		…	…
Age group	Under 15	950	19.18	Reference		Reference	…
	16 to 64	1887	38.11	1.15 (.91, 1.44)	.24	1.01 (.8, 1.27)	.95
	65 to 84	1606	32.43	2.14 (1.72, 2.67)	<.001	1.66 (1.32, 2.08)	<.001
	85 and above	505	10.20	6.53 (5.05, 8.44)	<.001	4.67 (3.6, 6.04)	<.001
	Unknown	<5	0.08	…		…	…
Age	…	…	…	1.02 (1.02, 1.02)	<.001	1.02 (1.01, 1.02)	<.001
Age <15	…	3998	…	1.04 (.99, 1.10)	.112	1.04 (.99, 1.10)	.112
Age ≥15	…	950	…	1.04 (1.03, 1.04)	<.001	1.03 (1.03, 1.04)	<.001
Ethnicity	White	3895	78.66	Reference		Reference	…
	Asian	296	5.98	0.77 (.56, 1.05)	.10	0.88 (.63, 1.21)	.42
	Black	116	2.34	0.74 (.45, 1.22)	.24	0.85 (.51, 1.42)	.54
	Mixed	59	1.19	0.87 (.45, 1.68)	.68	1.06 (.55, 2.06)	.86
	Other	33	0.67	1.02 (.44, 2.36)	.96	1.25 (.54, 2.89)	.60
	Unknown	553	11.17	…		…	…
IMD^a^	1	746	15.06	Reference		Reference	…
	2	638	12.99	0.86 (.66, 1.11)	.25	0.93 (.71, 1.23)	.63
	3	692	13.97	0.89 (.69, 1.14)	.35	0.92 (.70, 1.21)	.56
	4	680	13.73	0.94 (.73, 1.21)	.62	1.10 (.84, 1.43)	.48
	5	618	12.48	1.08 (.84, 1.39)	.56	1.07 (.81, 1.41)	.63
	Unknown	1578	31.87	…		…	…
Lineage^b^	M1_global_	530	10.70	Reference		Reference	…
	M1_13SNP_	29	0.59	0.40 (.12, 1.35)	.14	0.47 (.14, 1.59)	.23
	M1_23SNP_	19	0.38	0.41 (.09, 1.8)	.24	0.23 (.03, 1.72)	.15
	M1_UK_	778	15.71	1.13 (.87, 1.47)	.37	1.12 (.85, 1.47)	.42
	Unknown	3596	72.62	…		…	…

Multivariate	Total 1356		*P*-value	Mortality, 7d, Adjusted OR (95% CI)	*P*-value
Sex	Male	Reference	…	Reference	
	Female	1.21 (.93, 1.57)	.15	1.28 (.97, 1.68)	.08
Age (y)	…	1.02 (1.02, 1.03)	<.001	1.02 (1.01, 1.02)	.00
Lineage	M1_global_	Reference	…	Reference	
	M1_13SNP_	0.42 (.12, 1.43)	.16	0.51 (.15, 1.73)	.28
	M1_23SNP_	0.37 (.08, 1.7)	.20	0.21 (.03, 1.62)	.13
	M1_UK_	1.12 (.86, 1.47)	.40	1.12 (.84, 1.48)	.44

Abbreviations: CI, confidence interval; OR, odds ratio.

^a^IMD, Index of Multiple Deprivation.

^b^Univariate analysis limited to those with lineage assigned (*n* = 1356).

**Figure 1.**
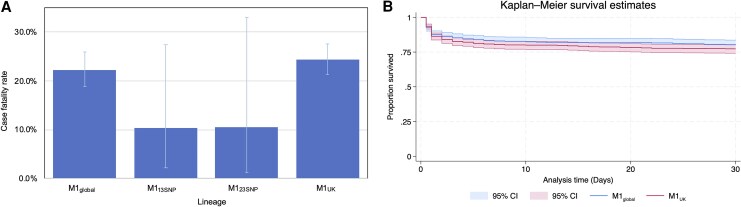
30-d case fatality and survival by *emm*1 lineage. *A*, Columns show 30-d case fatality rate, error bars represent 95% CI. Data are from 1356 patients for whom lineage was assigned and with followup data. Denominators for each group are M1_global_ (*n* = 530); M1_13SNPs_ (*n* = 29); M1_23SNPs_ (*n* = 19); M1_UK_ (*n* = 778). *B*, 30-d survival of M1_global_ and M1_UK_ iGAS patients. Shaded regions show 95% CI. Number in each group M1_global_ (*n* = 530); M1_UK_ (*n* = 778). Those who died (day 7 to day 1) inclusive were recorded as dying at time zero 0. Those who died on the day of the sample were recorded as dying at time 0.5 d. Abbreviations: CI, confidence interval; iGAS, invasive group A streptococcus; SNPs, single nucleotide polymorphism; UK, United Kingdom.

When considering only the 1356 *emm*1 isolates that were assigned to a lineage (2010–2020), the 30-day CFR was 22.3% (118/530, 95% CI: 18.8%, 26.0%) for iGAS caused by M1_global_ and 24.4% (190/778, 95% CI: 21.4%, 27.6%) for iGAS caused by M1_UK_, a difference that was not significant. The 30-day CFR for iGAS caused by the intermediate lineages was 10.3% (M1_13SNP_) and 10.5% (M1_23SNP_), although the denominators in these groups were small and differences were not significant (3/29 and 2/19 deaths, respectively) (Figure 1*A*). The 7-day case fatality rates followed a similar pattern (Supplementary Table 1).

Kaplan–Meier analysis revealed no difference in survival between M1_global_ and M1_UK_ cases over 30 days (Figure 1*B*) (*P* = .24), even when analyzed by age group (Supplementary Figure 2). Of note, almost all deaths beyond 3 days of sampling were seen in the older age groups only (Supplementary Figure 2).

### Univariate and Multivariate Analyses

Univariate analysis of the entire study cohort of *emm1* cases from December 2009 to July 2022 indicated an increase in risk of 30-day mortality from iGAS among females compared with males (OR 1.16, 95% CI: 1.01–1.33, *P*, .04). Age had a greater impact on mortality, with those aged 65–84 having a 2-fold, and those aged 85 and older having a 6.5-fold increased risk of death compared with children (Table 1). Indeed, every year of age increased the odds of dying in 30 days by 4% amongst those over 15 years of age. Although no effect of ethnicity or deprivation was observed overall, analysis by age subgroup pointed to an increased risk of 7- and 30-day mortality among Black (OR 2.94, 95% CI: 1.49–5.70, *P* < .001) and Asian (OR 1.76, 95% CI: 1.01–3.08, *P*, .05) children under 15 years (Supplementary Table 2).

To determine whether M1_UK_ was associated with increased risk of death, multivariate analysis was performed for the subcohort with complete data for lineage, and adjusted for age and sex (Table 1). After adjusting for these factors, age was the only factor conferring an increased risk of 30-day and 7-day mortality. Considering the cohort of iGAS patients with complete data, *emm*1 lineage did not have a significant effect.

### Time to Death Analysis

**Figure 2.**
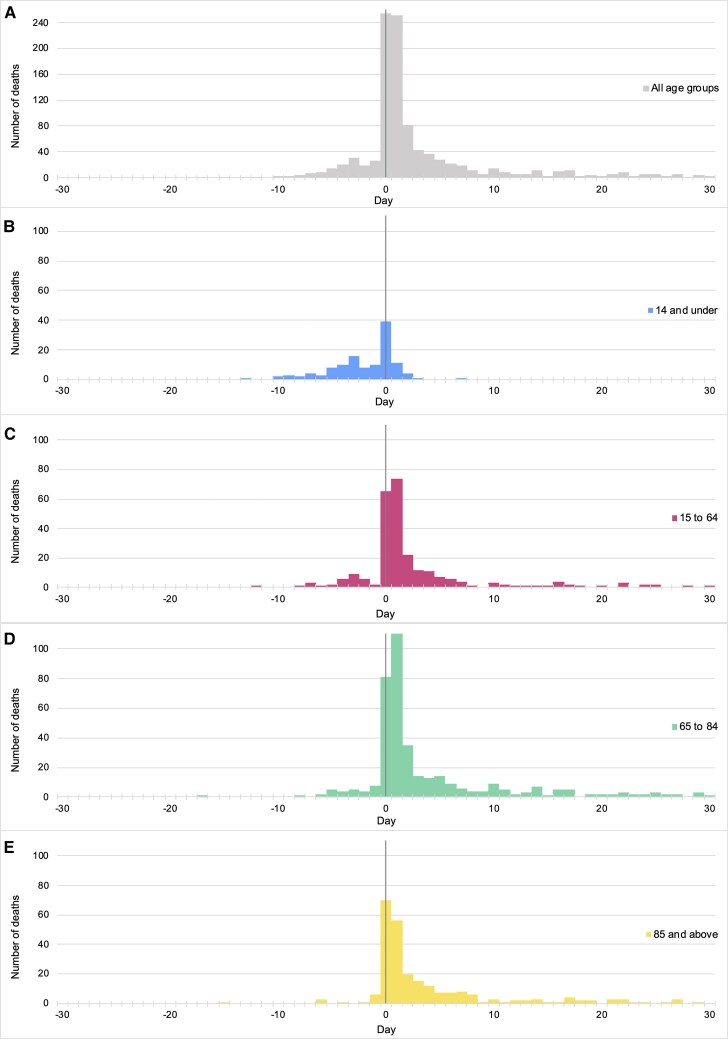
Time between diagnostic sample and death in different age groups. Each panel shows deaths from *emm*1 iGAS in all age groups (*A*); in children aged 14y and under (*B*); in adults aged 15–64 y (*C*); in adults aged 65y–84y (*D*); and in adults aged 85y and over (*E*). *Y* axes show the number of deaths for each sublineage. *X* axes show the number of days since the date of diagnostic sample that yielded *Streptococcus pyogenes*. Abbreviation: iGAS, invasive group A streptococcus.

Considering all deaths in the study cohort of *emm*1 *S. pyogenes* iGAS cases (Supplementary Table 3), we analyzed the time from diagnostic sample being obtained to death (using an extended period, from −30 days to +30 days from date of sample, *n* = 1060 deaths, one of unknown age). Strikingly, among children under 15 years who died, a majority (56.3%, 76/135) died before any sample was taken, 87.4% (118/135) had died by the day of sampling, and 95.6% (129/135) had died by the day after sampling; only 2 deaths in children were observed after 2 days of sample being taken (Figure 2). Even among older age groups, most deaths had occurred by one day after a sample had been taken for culture (15–64y: 65%, 182/280; 65–84 years: 57.5%, 226/393; 85years and above: 55%, 138/251) (Figure 2). When considering only those deaths where *emm*1 isolates were assigned to a lineage, no difference between M1_global_ and M1_UK_ with regard to time to death was observed (Supplementary Figure 3).

## DISCUSSION

We set out to determine the CFR of iGAS caused by *emm*1 *S. pyogenes* and determine the CFR due to emergent lineage M1_UK_ compared with ancestral *emm*1 strains. We found the average case fatality rate (CFR) associated with M1_UK_ iGAS in the pre-COVID19 pandemic period to be 24.4%; this was not significantly different from the CFR for iGAS caused by M1_global_ isolates (22.3%). The very high CFR associated with *emm*1 *S. pyogenes* was impressive but is similar to that reported from the United States for *emm*1 iGAS [36]. Mortality for the intermediate *emm*1 lineages was 10%.

Although M1_UK_ was not significantly more lethal than M1_global_ in the cohort examined, enhanced fitness for transmission is believed to underlie its increasing dominance. Whether this is linked to the increased production of superantigen SpeA or other metabolic attributes is unclear [37]. A number of studies report that M1_UK_ isolates display minimal genetic change over a fixed time period, indicating genomic stability despite widespread transmission [7, 16, 18] with dominance even in non-invasive infections [38]. As population immunity evolves, it may be that any advantage dwindles. Given that M1_UK_ dominated many of the reported iGAS upsurges in the post-pandemic period [6–8, 14, 16], our findings go some way to explaining the observed numbers of fatalities in such upsurges [14, 39]. The CFR (10%) observed following iGAS with intermediate lineages M1_13SNP_ and M1_23SNP_ was surprising, albeit that these groups comprised just 48 patients, making statistical comparison challenging. The moleculo-epidemiological data suggest that these intermediate lineages do not share the fitness to cause severe invasive infection that is seen in the major dominant *emm*1 lineages, despite M1_23SNP_ isolates expressing SpeA to the same level as M1_UK_ strains [37]. Arising around 2002 and 2006, respectively, as antecedents of M1_UK_, these intermediate lineages are no longer detectable in England [16, 38], and the current cohort provides the only available outcome data.

Univariate analysis indicated non-white ethnicity in children under 15 years to be associated with increased CFR, but differences were not seen among adults. This disparity will be examined in future studies that examine parent and caregiver experience of children's illness and healthcare access, and examine patterns in other infections. Although we were able to include ethnicity and markers of deprivation in our analysis, we were unable to include comorbidity, which can have a major influence on outcome in many bacterial infections including iGAS.

The study had a number of limitations common to all retrospective analyses. It is possible that iGAS outcome more generally may have improved over time due to changes in sepsis management, for example, more widespread use of clindamycin, potentially impacting results. Our study was limited by an inability to balance cases from each lineage over time, due to the very rapid expansion of M1_UK_ in England between 2010 and 2015 [18], and by the number of iGAS cases for which lineage had been assigned. Our lineage-specific study is unlikely to have been affected by the COVID-19 pandemic, as the vast majority of *emm*1 iGAS in 2020 was in Q1 only, after which *emm*1 iGAS was rare. We deliberately excluded the post-COVID-19 iGAS upsurge period in 2022–2023, to avoid the impact of any population immunity gap and respiratory viruses on outcomes [15, 16].

Our study did not demonstrate a substantial difference in CFR between the two major *emm*1 lineages but lacked power to demonstrate a more modest (5%) difference; such a study might require >1000 cases in each group. Formal comparison of CFR would be valuable from other regions where both lineages currently co-exist, using agreed standard definitions to allow meta-analysis. Active surveillance of *emm*1 from the Centers for Disease Control and Prevention from 10 US states 2015–2021 reported the CFR of M1_UK_ to be 22% and non-M1_UK_ to be 14.9%, a difference that was not significant [36]. The study had a limited sample size for M1_UK_ cases with a 20-fold imbalance between cohorts, and non-M1_UK_ cases may have included intermediate lineages [36]. Taking both studies together, there is a trend toward greater mortality associated with M1_UK,_ but neither study found a significant difference.

A striking observation was the rapidity of death caused by *emm*1 iGAS infections regardless of lineage, a feature of iGAS that has been highlighted before [22]. A majority of deaths occurred within just one day of a sample being taken for culture, the earliest point that culture-based diagnosis might be obtained. Indeed, in children, >50% deaths occurred before any sample was taken, underlining a need for case-based reviews to identify what interventions might improve outcomes, including implementation of rapid diagnostic tests, value of clinical severity scores, and importance of enhancing prevention of transmission during outbreaks, including vaccine introduction. Few bacterial infections progress as swiftly as iGAS in previously healthy people, contributing to legal proceedings seeking accountability when deaths occur [40]. The rapidity of deaths has implications for clinical trial design: for *emm*1 iGAS, waiting for culture-based diagnostic confirmation may make any intervention futile, as most deaths attributable to *S. pyogenes* will already have happened.

Emergence of a new variant of any pathogen should prompt rapid evaluation of the potential for harm; while this is well-understood for viruses of pandemic potential, the principle is applicable to bacteria and other microorganisms as well. This is true not only for bacterial lineages that harbor specific antimicrobial resistance traits, but also acutely relevant for bacteria of high pathogenicity such as *S. pyogenes*, for which there are no vaccines. For rare infections, evaluation of CFR may require a multi-country meta-analysis. As an exclusively human pathogen, a better understanding of the severity that can be attributed to different lineages of *S. pyogenes* will be of great value in planning for, and managing, the public's health.

## Supplementary Material

ciaf492_Supplementary_Data

## Supplementary Data

Supplementary materials are available at *Clinical Infectious Diseases* online. Consisting of data provided by the authors to benefit the reader, the posted materials are not copyedited and are the sole responsibility of the authors, so questions or comments should be addressed to the corresponding author.
