# Supplementary material for: Mortality Among Patients With Invasive Group A Streptococcal Infections Caused by the M1UK Lineage: A Retrospective Cohort Study in England and Wales
Source: Clin Infect Dis. 2025 Oct 16;81(5):e277–84. doi: 10.1093/cid/ciaf492 (PMC12728285; doi:10.1093/cid/ciaf492)
Supplement: ciaf492_Supplementary_Data [file ciaf492_supplementary_data.docx]

**Supplementary Material for**

Mortality among patients with invasive group A streptococcal infections caused by the M1_UK_ lineage: a retrospective cohort study in England and Wales

**Li HK, Zhu N, et al.**

**Supplementary Tables**

Table S1 Case fatality rate by age group. Page 2

Table S2 Univariate analysis by age group Page 3

Table S3 Case fatality by demographic sub group Page 6

**Supplementary Figures**

Figure S1 Emm1 iGAS cases and isolates assigned to lineage over time**.** Page 7

Figure S2 30-day survival of M1_global_ and M1_UK_ iGAS cases by age group Page 8

Figure S3 Time between sample and death by lineage Page 9

**Table S1 Case fatality rate by age group**

| All age groups |  |  |  |  |  |
| --- | --- | --- | --- | --- | --- |
|  | **All** | **M1_global_** | **M1_13SNP_** | **M1_23SNP_** | **M1_UK_** |
| Total | 4,952 | 530 | 29 | 19 | 778 |
| Died, 30 days | 1,043 | 118 | 3 | 2 | 190 |
| CFR, 30 days (95% CI) | 0.211 [0.199, 0.222] | 0.223 [0.188, 0.260] | 0.103 [0.022, 0.274] | 0.105 [0.013, 0.331] | 0.244 [0.214, 0.276] |
| Died, 7 days | 901 | 104 | 3 | 1 | 167 |
| CFR, 7 days (95% CI) | 0.182 [0.171, 0.193] | 0.196 [0.163, 0.233] | 0.103 [0.022, 0.274] | 0.053 [0.001, 0.260] | 0.215 [0.186, 0.245] |
| Under 15 years |  |  |  |  |  |
|  | **All** | **M1_global_** | **M1_13SNP_** | **M1_23SNP_** | **M1_UK_** |
| Total | 950 | 100 | 5 | 5 | 149 |
| Died, 30 days | 124 | 17 | 0 | 0 | 19 |
| CFR, 30 days (95% CI) | 0.131 [0.110, 0.154] | 0.170 [0.102, 0.258] | 0.000 [0.000, 0.522] | 0.000 [0.000, 0.522] | 0.128 [0.079, 0.192] |
| Died, 7 days | 124 | 17 | 0 | 0 | 19 |
| CFR, 7 days (95% CI) | 0.131 [0.110, 0.154] | 0.170 [0.102, 0.258] | 0.000 [0.000, 0.522] | 0.000 [0.000, 0.522] | 0.128 [0.079, 0.192] |
| 15 to 64 years |  |  |  |  |  |
|  | **All** | **M1_global_** | **M1_13SNP_** | **M1_23SNP_** | **M1_UK_** |
| Total | 1887 | 200 | 12 | 5 | 296 |
| Died, 30 days | 277 | 26 | 0 | 0 | 52 |
| CFR, 30 days (95% CI) | 0.147 [0.131, 0.164] | 0.130 [0.087, 0.185] | 0.000 [0.000, 0.265] | 0.000 [0.000, 0.522] | 0.176 [0.134, 0.224] |
| Died, 7 days | 248 | 22 | 0 | 0 | 50 |
| CFR, 7 days (95% CI) | 0.131 [0.116, 0.148] | 0.110 [0.070, 0.162] | 0.000 [0.000, 0.265] | 0.000 [0.000, 0.522] | 0.169 [0.128, 0.217] |
| 65 to 84 years |  |  |  |  |  |
|  | **All** | **M1_global_** | **M1_13SNP_** | **M1_23SNP_** | **M1_UK_** |
| Total | 1606 | 184 | 10 | 5 | 239 |
| Died, 30 days | 391 | 50 | 2 | 0 | 72 |
| CFR, 30 days (95% CI) | 0.243 [0.223, 0.265] | 0.272 [0.209, 0.342] | 0.200 [0.025, 0.556] | 0.000 [0.000, 0.522] | 0.234 [0.175, 0.302] |
| Died, 7 days | 320 | 43 | 2 | 0 | 63 |
| CFR, 7 days (95% CI) | 0.199 [0.180, 0.220] | 0.301 [0.244, 0.364] | 0.200 [0.025, 0.556] | 0.000 [0.000, 0.522] | 0.264 [0.209, 0.324] |
| 85 years and above |  |  |  |  |  |
|  | **All** | **M1_global_** | **M1_13SNP_** | **M1_23SNP_** | **M1_UK_** |
| Total | 505 | 46 | 2 | 4 | 93 |
| Died, 30 days | 250 | 25 | 1 | 2 | 47 |
| CFR, 30 days (95% CI) | 0.495 [0.451, 0.540] | 0.543 [0.390, 0.691] | 0.500 [0.013, 0.987] | 0.500 [0.068, 0.932] | 0.478 [0.329, 0.631] |
| Died, 7 days | 208 | 22 | 1 | 1 | 35 |
| CFR, 7 days (95% CI) | 0.412 [0.369, 0.456] | 0.505 [0.400, 0.611] | 0.500 [0.013, 0.987] | 0.250 [0.006, 0.806] | 0.376 [0.278, 0.483] |

**Table S2 Univariate analysis by age group**

|  |  | Number (N) | Percentage (%) | Mortality, 30 days, odds ratio (95% CI) | p-value | Mortality, 7 days, odds ratio (95% CI) | p-value |
| --- | --- | --- | --- | --- | --- | --- | --- |
| Under 15 |  |  |  |  |  |  |  |
| Sex | Male | 500 | 52.63 | Reference |  | Reference |  |
|  | Female | 440 | 46.32 | 1.08 (0.74, 1.57) | 0.71 | 1.08 (0.74, 1.57) | 0.71 |
|  | Unknown | 10 | 1.05 |  |  |  |  |
| Ethnicity | White | 618 | 65.05 | Reference |  | Reference |  |
|  | Asian | 114 | 12.00 | 1.76 (1.01, 3.08) | 0.05 | 1.76 (1.01, 3.08) | 0.05 |
|  | Black | 52 | 5.47 | 2.94 (1.49, 5.79) | <0.001 | 2.94 (1.49, 5.79) | <0.001 |
|  | Mixed | 36 | 3.79 | 2.13 (0.89, 5.05) | 0.09 | 2.13 (0.89, 5.05) | 0.09 |
|  | Other | 9 | 0.95 | 2.52 (0.51, 12.38) | 0.26 | 2.52 (0.51, 12.38) | 0.26 |
|  | Unknown | 121 | 12.74 |  |  |  |  |
| IMD | 1 | 206 | 21.68 | Reference |  | Reference |  |
|  | 2 | 116 | 12.21 | 0.47 (0.21, 1.07) | 0.07 | 0.47 (0.21, 1.07) | 0.07 |
|  | 3 | 116 | 12.21 | 1.25 (0.66, 2.34) | 0.50 | 1.25 (0.66, 2.34) | 0.50 |
|  | 4 | 91 | 9.58 | 0.87 (0.41, 1.84) | 0.72 | 0.87 (0.41, 1.84) | 0.72 |
|  | 5 | 76 | 8.00 | 0.54 (0.22, 1.37) | 0.20 | 0.54 (0.22, 1.37) | 0.20 |
|  | Unknown | 345 | 36.32 |  |  |  |  |
| Lineage | M1_global_ | 100 | 10.53 | Reference |  | Reference |  |
|  | M1_13SNP_ | 5 | 0.53 | Empty |  | Empty |  |
|  | M1_23SNP_ | 5 | 0.53 | Empty |  | Empty |  |
|  | M1_UK_ | 149 | 15.68 | 0.71 (0.35, 1.45) | 0.35 | 0.71 (0.35, 1.45) | 0.35 |
|  | Unknown | 691 | 72.74 |  |  |  |  |
| 15 to 64 |  |  |  |  |  |  |  |
| Sex | Male | 992 | 52.57 | Reference |  | Reference |  |
|  | Female | 885 | 46.9 | 0.97 (0.75, 1.26) | 0.83 | 1.04 (0.79, 1.36) | 0.78 |
|  | Unknown | 10 | 0.53 |  |  |  |  |
| Ethnicity | White | 1,453 | 77.00 | Reference |  | Reference |  |
|  | Asian | 109 | 5.78 | 0.90 (0.5, 1.6) | 0.71 | 0.93 (0.51, 1.69) | 0.81 |
|  | Black | 55 | 2.91 | 0.48 (0.17, 1.34) | 0.16 | 0.40 (0.12, 1.28) | 0.12 |
|  | Mixed | 17 | 0.90 | 1.30 (0.37, 4.58) | 0.68 | 1.47 (0.42, 5.16) | 0.55 |
|  | Other | 20 | 1.06 | 1.52 (0.5, 4.6) | 0.46 | 1.71 (0.57, 5.18) | 0.34 |
|  | Unknown | 233 | 12.35 |  |  |  |  |
| IMD | 1 | 296 | 15.69 | Reference |  | Reference |  |
|  | 2 | 258 | 13.67 | 0.98 (0.63, 1.54) | 0.94 | 1.11 (0.7, 1.77) | 0.66 |
|  | 3 | 257 | 13.62 | 0.63 (0.38, 1.02) | 0.06 | 0.52 (0.3, 0.91) | 0.02 |
|  | 4 | 258 | 13.67 | 0.72 (0.45, 1.16) | 0.18 | 0.83 (0.51, 1.36) | 0.47 |
|  | 5 | 215 | 11.39 | 0.80 (0.49, 1.30) | 0.37 | 0.95 (0.58, 1.58) | 0.86 |
|  | Unknown | 603 | 31.96 |  |  |  |  |
| Lineage | M1_global_ | 200 | 10.60 | Reference |  | Reference |  |
|  | M1_13SNP_ | 12 | 0.64 | Empty |  | Empty |  |
|  | M1_23SNP_ | 5 | 0.26 | Empty |  | Empty |  |
|  | M1_UK_ | 296 | 15.69 | 1.43 (0.86, 2.37) | 0.17 | 1.64 (0.96, 2.81) | 0.07 |
|  | Unknown | 1,374 | 72.81 |  |  |  |  |
| 65 to 84 |  |  |  |  |  |  |  |
| Sex | Male | 905 | 56.35 | Reference |  | Reference |  |
|  | Female | 696 | 43.34 | 1.06 (0.84, 1.33) | 0.64 | 1.10 (0.86, 1.40) | 0.46 |
|  | Unknown | 5 | 0.31 |  |  |  |  |
| Ethnicity | White | 1,380 | 85.93 | Reference |  | Reference |  |
|  | Asian | 63 | 3.92 | 0.84 (0.45, 1.56) | 0.58 | 0.89 (0.46, 1.73) | 0.73 |
|  | Black | 7 | 0.44 | 0.54 (0.06, 4.47) | 0.57 | 0.70 (0.08, 5.85) | 0.74 |
|  | Mixed | 6 | 0.37 | 0.64 (0.07, 5.53) | 0.69 | 0.84 (0.10, 7.23) | 0.88 |
|  | Other | 4 | 0.25 | 1.07 (0.11, 10.35) | 0.95 | 1.40 (0.15, 13.54) | 0.77 |
|  | Unknown | 146 | 9.09 |  |  |  |  |
| IMD | 1 | 179 | 11.15 | Reference |  | Reference |  |
|  | 2 | 202 | 12.58 | 0.64 (0.41, 1.02) | 0.06 | 0.95 (0.57, 1.59) | 0.85 |
|  | 3 | 245 | 15.26 | 0.61 (0.39, 0.95) | 0.03 | 0.88 (0.53, 1.44) | 0.60 |
|  | 4 | 255 | 15.88 | 0.79 (0.52, 1.21) | 0.28 | 1.27 (0.79, 2.02) | 0.33 |
|  | 5 | 242 | 15.07 | 0.70 (0.45, 1.08) | 0.11 | 0.91 (0.56, 1.5) | 0.72 |
|  | Unknown | 483 | 30.07 |  |  |  |  |
| Lineage | M1_global_ | 184 | 11.46 | Reference |  | Reference |  |
|  | M1_13SNP_ | 10 | 0.62 | 0.67 (0.14, 3.26) | 0.62 | 0.82 (0.17, 4.01) | 0.81 |
|  | M1_23SNP_ | 5 | 0.31 | Empty |  | Empty |  |
|  | M1_UK_ | 239 | 14.88 | 1.16 (0.75, 1.77) | 0.51 | 1.17 (0.75, 1.83) | 0.48 |
|  | Unknown | 1,168 | 72.73 |  |  |  |  |
| 85 and above |  |  |  |  |  |  |  |
| Sex | Male | 210 | 41.58 | Reference |  | Reference |  |
|  | Female | 294 | 58.22 | 1.44 (1.01, 2.06) | 0.04 | 1.21 (0.84, 1.74) | 0.30 |
|  | Unknown | 1 | 0.2 |  |  |  |  |
| Ethnicity | White | 444 | 87.92 | Reference |  | Reference |  |
|  | Asian | 10 | 1.98 | 0.69 (0.19, 2.48) | 0.57 | 1.00 (0.28, 3.58) | 1.00 |
|  | Black | 2 | 0.40 | 1.04 (0.06, 16.68) | 0.98 | 1.49 (0.09, 24.05) | 0.78 |
|  | Mixed | 0 |  | Empty |  | Empty |  |
|  | Other | 0 |  | Empty |  | Empty |  |
|  | Unknown | 49 | 9.70 |  |  |  |  |
| IMD | 1 | 65 | 12.87 | Reference |  | Reference |  |
|  | 2 | 62 | 12.28 | 0.91 (0.45, 1.82) | 0.79 | 0.69 (0.34, 1.4) | 0.30 |
|  | 3 | 73 | 14.46 | 1.18 (0.6, 2.3) | 0.64 | 1.07 (0.55, 2.1) | 0.83 |
|  | 4 | 76 | 15.05 | 0.78 (0.4, 1.52) | 0.48 | 0.80 (0.41, 1.57) | 0.52 |
|  | 5 | 85 | 16.83 | 1.61 (0.83, 3.09) | 0.16 | 0.99 (0.52, 1.89) | 0.97 |
|  | Unknown | 144 | 28.51 |  |  |  |  |
| Lineage | M1_global_ | 46 | 9.11 | Reference |  | Reference |  |
|  | M1_13SNP_ | 2 | 0.40 | 0.84 (0.05, 14.26) | 0.90 | 1.09 (0.06, 18.51) | 0.95 |
|  | M1_23SNP_ | 4 | 0.79 | 0.84 (0.11, 6.49) | 0.87 | 0.36 (0.04, 3.76) | 0.40 |
|  | M1_UK_ | 93 | 18.42 | 0.86 (0.42, 1.74) | 0.67 | 0.66 (0.32, 1.35) | 0.25 |
|  | Unknown | 360 | 71.29 |  |  |  |  |

**Table S3 Case fatality rate by demographic sub group**

|  |  | Number (N) | Died, 30 days | Died, 7 days |
| --- | --- | --- | --- | --- |
| Total |  | 4,952 | 1043 | 901 |
|  |  |  |  |  |
| Sex | Male | 2,608 | 521 | 448 |
|  | Female | 2,317 | 519 | 450 |
|  | Unknown | 27 | <5 | <5 |
|  |  |  |  |  |
| Age group | Under 15 | 950 | 124 | 124 |
|  | 16 to 64 | 1,887 | 277 | 248 |
|  | 65 to 84 | 1,606 | 391 | 320 |
|  | 85 and above | 505 | 250 | 208 |
|  | Unknown | <5 | <5 | <5 |
|  |  |  |  |  |
| Ethnicity | White | 3,895 | 813 | 691 |
|  | Asian | 296 | 50 | 47 |
|  | Black | 116 | 19 | 18 |
|  | Mixed | 59 | 11 | 11 |
|  | Other | 33 | 7 | 7 |
|  | Unknown | 553 | 143 | 127 |
|  |  |  |  |  |
| IMD | 1 | 746 | 165 | 136 |
|  | 2 | 638 | 125 | 110 |
|  | 3 | 692 | 139 | 118 |
|  | 4 | 680 | 143 | 134 |
|  | 5 | 618 | 145 | 119 |
|  | Unknown | 1,578 | 326 | 284 |
|  |  |  |  |  |
| Lineage | M1_global_ | 530 | 118 | 104 |
|  | M1_13SNP_ | 29 | <5 | <5 |
|  | M1_23SNP_ | 19 | <5 | <5 |
|  | M1_UK_ | 778 | 190 | 167 |
|  | Unknown | 3,596 | 730 | 626 |

**Supplementary Figure S1**

**A.**

**B.**

**Supplementary Figure S1 Emm1 iGAS cases and isolates assigned to lineage over time.** Total iGAS cases associated with *emm*1 *S. pyogenes* isolates per month referred to the reference laboratory are shown over the study period Dec 2009-July 2022. UK lockdown onset indicated with triangle (A). Lineage (%) of *emm*1 isolates that were genotyped and used in this study (B). These included previously sequenced *emm*1 iGAS isolates from March-May 2013; the 14 month period April 2014-May 2015; and March-May 2016 (Lynskey NN et al, Lancet Infect Dis 2019; 19(11):1209-1218 and Kapatai G, et al. PeerJ 2017; 5: e3226.) For 2010 and 2020, all available *emm*1 iGAS isolates were included and genotyped by allele-specific PCR.

**Supplementary Figure S2**

1. **B.**

**Supplementary Figure S2. Kaplan Meier survival plot showing 30-day survival of M1_global_ and M1_UK_ iGAS cases by age group.**

Left panel (A) shows M1_global_ iGAS cases. Right panel (B) shows M1_UK_ iGAS cases. Age group indicated by line colour. Children aged under 15y blue; Adults aged 15y - 64y red; Adults aged 65y -8y4 green; Adults aged over 85y yellow.

**Supplementary Figure S3**

**Supplementary Figure S3. Time between diagnostic sample and death by lineage.** Each panel shows deaths from each *emm*1 lineage as labelled from top to bottom: M1_global_ (blue); M1_13SNPs_ (light blue); M1_23SNPs_ (light pink); M1_UK_ (red). Y axes show numbers of deaths for each sublineage. X axes show number of days since date of diagnostic sample that yielded *S. pyogenes*.
